# Molecular characterization of blood type *A*, *B*, and *C (AB)* in domestic cats and a *CMAH* genotyping scheme

**DOI:** 10.1371/journal.pone.0204287

**Published:** 2018-09-20

**Authors:** Alexandra Kehl, Kevin Heimberger, Ines Langbein-Detsch, Sabine Boehmer, Karthik Raj, Elisabeth Mueller, Urs Giger

**Affiliations:** 1 Laboklin, Bad Kissingen, Germany; 2 Section of Medical Genetics (PennGen), University of Pennsylvania, Philadelphia, Pennsylvania, United States of America; Universidade de Sao Paulo, BRAZIL

## Abstract

In domestic cats, the *AB* blood group system consists of the three types *A*, *B*, and *C* (usually called *AB*), which vary in frequency among breeds and geographic regions. Mismatches cause acute hemolytic transfusion reactions and hemolysis of the newborn due to the presence of naturally occurring *anti-A* alloantibodies. Cytidine monophosphate-N-acetylneuraminic acid hydroxylase (CMAH) converts N-acetylneuraminic acid (type *B*) to N-glycolylneuraminic acid (type *A*), and type *C* erythrocytes express both antigens. We examined the feline *CMAH* coding regions and genotyped cats to characterize type *A*, *B*, and *C* animals. Of 421 phenotypically typed cats, 60% were *A*, 35% *B* and 5% *C*. Among the 70 cats for which the *CMAH* coding region was sequenced, 13 new variants were identified in addition to 16 of the previously reported 18 variants. The *CMAH* variant c.268T>A is seen in type *B* cats of most breeds, and the variant c.179G>T results in type *B* in Turkish breeds. The variants c.1322delT and c.933delA cause frameshifts with early stop codons and thereby type *B* in some Ragdolls and domestic shorthair cats, respectively. Protein modeling with PROVEAN affirmed their deleterious effects. No type *A* and *C* cats had more than one allele with one of the above variants. Variant analysis of three SNVs (c.142G>A, c.268T>A and Δ-53) and blood typing of an additional 351 typed cats showed complete phenotype-genotype concordance. In conclusion, the three *CMAH* variants c.179G>T, c.268T>A and c.1322delT are the main reasons for the defective NeuGc synthesis causing blood type *B* in domestic purebred and non-pedigreed cats. Together with the variant c.364C>T for type *C* in Ragdolls they offer a molecular screening scheme for clinical diagnostics to assure blood type compatibility.

## Introduction

In purebred and non-pedigreed domestic cats (*felis catus*), the *AB* blood group system with type *A*, *B*, and *C* (usually called *AB*) is of main concern, as blood incompatibilities can result in acute hemolytic transfusion reactions and hemolysis of the newborn (neonatal isoerythrolysis) due to naturally occurring alloantibodies [1–3]. The enzyme cytidine monophosphate-N-acetylneuraminic acid hydroxylase (CMAH; EC 1.14.18.2) converts sialic acid N-acetylneuraminic acid (NeuAc; type *B* antigen) to N-glycolylneuraminic acid (NeuGc; type *A* antigen) [3]. Depending on the blood type, plasma contains strong naturally occurring *anti-A* and sometimes *anti-B* antibodies against the other red blood cell (RBC) antigens [4]. Cats with blood type *C* express both NeuAc and NeuGc in varied amounts and have no *anti-A* and *anti-B* alloantibodies. Furthermore, their RBCs agglutinate with antibodies against both NeuGc and NeuAc [5, 6].

The frequencies of type *A*, *B*, and *C* vary among feline breeds and geographic regions [3, 7]. The *A* allele was determined to be dominant to the *b* allele, and the *a*^*c*^ allele (causing blood type *C*) was found to be separately inherited, being (co-) dominant to *b* and recessive to the *A* allele [5, 7]. Thus, the following genotypes can be observed: *A*/*A*, *A*/*b* and *A*/*a*^*c*^ for type *A*, *b/b* for type *B*, and *a*^*c*^*/ a*^*c*^ or *a*^*c*^*/b* for type *C*.

While good immunological typing assays are available for clinics and diagnostic laboratories [3], genotyping could also be used to determine the recessive *b* and *a*^*c*^ alleles in type *A* and *C* cats to confirm and predict blood types in offspring. In recent limited molecular genetic studies several variants in the *CMAH* gene were thought to be responsible for type *A*, *B*, and *C* [8–11]. The *b* allele was ascribed to two single nucleotide variants (SNVs) upstream of the open reading frame (ORF) of the *CMAH* gene (-371C>T and -217G>A), alongside an insertion/deletion of 18 bp in the 5´-untranslated region (UTR) 53 bp upstream (Δ-53) and four non-synonymous variants within the ORF (c.139C>T, c.142G>A, c.268T>A, and c.1603G>A) [8, 9]. However, there were many examples of phenotype-genotype discordances for cats with type *B* and *C* (personal unpublished results) [10, 11], making genotyping unreliable particularly for cats with type *B* and *C* blood, where genotyping would be most helpful. In a fourth study, additional variants were associated with a specific blood type as diplotypes and haplotypes, but not as individual variants [11]. The variant c.364C>T (p.Pro122Ser) was reported to cause type *C* in Ragdolls and some non-pedigreed cats, whereby the few type *C* cats from other breeds did not show this variant. It is speculated that the variant c.364C>T reduces the activity of CMAH leading to the presence of both NeuGc and NeuAc, but no biochemical studies have been performed [10]. These prior studies included a limited number of cats with unconfirmed phenotyping results; blood typing is done immunologically in clinics and diagnostic laboratories, and it is recommended to confirm type *B* and *C* results with back typing and or crossmatching by a reference laboratory [3, 12–14]. Additionally, the effects of the discovered variants were not studied by protein modeling, expression, and/or function experiments to determine which might be the variants impairing CMAH function and thus type *B* and *C* blood. A DNA screening scheme involving *CMAH* variants that could accurately determine the blood type within the feline *AB* blood group system would be highly desirable to complement the phenotypic tests. It should be noted that in humans a large deletion makes *CMAH* a pseudogene, which is the cause for the lack of NeuGc expression in all humans [15]. Similarly, in several species other than felides the *AB* blood group system has not been found to be clinically important [16, 17].

In the present study, we sequenced the exons and regulatory regions of the *CMAH* gene from 70 purebred and non-pedigreed domestic cats which had been carefully phenotyped as having type *A*, *B*, and *C* blood, assessed the non-synonymous variants by modeling, and genotyped an additional 351 blood typed cats. We discovered new variants associated with type *A*, *B*, and *C*, report their predicted effect on the enzyme, and are proposing a simple scheme with SNVs to accurately screen cats genetically for all three blood types in different breeds.

## Materials and methods

### Cats and blood samples

A total of 421 ethylenediaminetetraacetic acid (EDTA) blood samples from domestic cats of various breeds submitted to the veterinary laboratory of Laboklin GmbH in Bad Kissingen, Germany, between mid of 2016 and the end of 2017 for routine diagnostic testing were analyzed. Some samples from type *B* and *C* cats were specifically selected and some were specifically submitted for confirmatory blood typing, as the laboratory was known to pursue additional blood typing assays to confirm blood type *B* and *C*. Breed of cats were recorded. These studies used left-over blood samples submitted to the laboratory and were approved by the Institutional Animal Care and Use Committee at the University of Pennsylvania (#805736).

### Phenotypic blood typing methods

Utilizing EDTA anticoagulated blood kept chilled for < one week, routine blood typing was performed with an immunochromatographic strip method (Alvedia rapid-test [LabTest *A+B*], Limonest, France) according to the manufacturer’s instructions [13, 14, 18], and results of all cats were confirmed by an additional forward typing method and back typing. For the additional forward typing with a gel column method (NaCl Enzyme Test and Cold Agglutinins, BioRad, Hercules, CA, USA), lectin *Triticum vulgaris* (0.005 μg/ml; Sigma-Aldrich, Darmstadt, Germany) as *anti-B* and serum from type *B* cats as *anti-A* were used as previously described [5, 12]. In order to detect *anti-A* alloantibodies in type *B* cats back typing was performed using their plasma with erythrocytes of known type *A* and type *B* blood in a gel column assay as previously described [5, 12]. The plasma of type *B* cats (>3 months) elicits agglutination with type *A* erythrocytes, while the plasma of type *A* cats shows no or weak agglutination with type *B* erythrocytes. Finally, type *C* cats have neither *anti-A* nor *anti-B* alloantibodies. Results of blood typing, using the immunochromatographic strip test, and additional forward- and back-typing had to be concordant for a cat to be used for sequencing or genotyping in this study (one cat was excluded because of divergent results).

### Sequencing of feline *CMAH* and variant detection

Samples from 70 cats were used for sequencing of the 16 exons (exon 1a, 1b, and exons 2–15) and regulatory regions including 5´UTR, 3´UTR and splice sites of the *CMAH* gene. However, the untranslated exon upstream of exon 1a was not sequenced [8, 11]. Genomic DNA was isolated from EDTA anticoagulated blood with the MagnaPure 96 kit (Hoffmann-La Roche, Basel, Switzerland). Primers used for amplifying the feline *CMAH* gene were based upon prior publications [8, 11] or designed based upon the feline *CMAH* sequence from Ensembl (Felis_catus_6.2:B2) using Primer3 software (http://primer3.sourceforge.net) (S1 Table). Products were amplified with the FastStart PCR Master Kit (Hoffmann-La Roche) and sequenced by cycle sequencing using ABI 3730XL sequencing equipment (Eurofins Genomics Sequencing Services, Ebersberg, Germany). The sequences were compared to the *CMAH* sequence from Ensembl (Felis_catus_6.2:B2) and NCBI (Genbank No. EF127684.1 and Reference No. NC_018727.3) using the Basic Local Alignment Search Tool (BLAST). All molecular genetic variants were numbered from the start codon (ATG), located in exon 1a according to EF127684.1. except the variants in the 5’UTR of exon 1b were numbered using the alternate transcript (splice variant). Note that for comparison, all numbers have to be reduced by three when counting from the start codon in exon 1b [11].

### Genotyping assays for *CMAH* SNVs and bioinformatics

Genotyping for c.142G>A, c.268T>A and Δ-53 was performed using TaqMan SNP Assays (Applied Biosystems, Waltham, USA) using FastStart Essential DNA Probes Master and LightCycler 480 II (Hoffmann-La Roche). Results from sequencing and genotyping assays were summarized as diplotypes and compared to phenotypic blood typing results in order to establish relationships between specific DNA variants and blood types *A*, *B*, and *C*. The effects of amino acid exchanges caused by SNVs were analyzed by SIFT (http://sift.jcvi.org/) and PROVEAN (http://provean.jcvi.org). Domain analyses were performed with ProSite (https://prosite.expasy.org/).

## Results

### Phenotypic blood typing

A total of 421 purebred (belonging to 16 breeds) and non-pedigreed domestic cats were phenotypically and genotypically blood typed. There were 255 cats with blood type *A* (60%), 146 with type *B* (35%), and 20 with type *C* (5%) with the distributions by breeds shown in Table 1. Note that this is a biased feline breed distribution, and samples from type *B* and *C* cats are overrepresented in comparison to the frequencies of *B* and *C* cats in specific breeds and the non-pedigreed cat populations and geographic regions for the purpose of this study. Blood typing results were concordant by immunochromatographic strip and gel column blood typing techniques for all cats studied. And all type *B* cats had strong *anti-A* alloantibodies, while very few type *A* cats had *anti-B* alloantibodies. Type *C* cats had neither *anti-A* nor *anti-B* alloantibodies.

**Table 1:** Phenotype distributions for the *AB* blood group system of the 421 purebred and non-pedigreed cats sequenced or genotyped in this study.

Breed	Blood type of						Total
	Sequenced cats			SNV genotyped cats			
	*A*	*B*	*C*	*A*	*B*	*C*	
Abyssinian	1	1	0	0	0	0	2
Bengal	0	0	0	2	0	0	2
Birman	1	4	0	27	14	0	46
British Longhair	0	1	0	0	4	0	5
British Shorthair	1	4	1	5	55	0	66
Devon Rex	0	0	0	0	1	0	1
Domestic Shorthair	0	1	6	58	16	0	81
Highlander	0	0	0	0	1	0	1
Maine Coon	2	2	0	47	2	0	53
Neva Masquerade	1	3	0	6	0	0	10
Norwegian Forest	0	0	0	6	0	0	6
Oriental Shorthair	0	0	0	2	0	0	2
Persian	1	0	0	25	3	0	29
Ragdoll	1	14	12	33	12	0	72
Scottish Fold	1	2	0	4	2	0	9
Siberian	1	0	1	25	0	0	27
Turkish Angora	4	4	0	1	0	0	9
Total	14	36	20	241	110	0	421

### Variants in the *CMAH* gene

The exonic and regulatory regions of the feline *CMAH* gene were successfully amplified and sequenced including all 16 exons (exons 1a, 1b and 2–15) with intronic boundaries and the 5´- and 3´-UTR from all 70 of the above mentioned purebred and non-pedigreed domestic cats that were sequenced (Table 1). The feline reference sequence (Felis_catus_6.2:B2) together with other published sequences of the type *A* cat (GenBank: EF127684.1 and Reference. NC_018727.3) [8, 10] were used as consensus sequence, diplotype, and haplotype for the wildtype *A* coding and regulatory sequences.

When comparing these sequences with *CMAH* sequences from 70 cats generated here, a total of 29 variants were identified in the coding and upstream regulatory regions of the *CMAH* gene (S1 Fig and S2 Table). These variants included 14 non-synonymous SNVs (nsSNVs; Table 2), 12 synonymous SNVs (sSNVs; S2 Table), and three indel variants (two frameshifts leading to stop codons; Table 2). Eight nsSNVs, the deletion Δ-53, and seven sSNVs were previously described (Table 2 and S2 Table) [8–11]. Only two previously described sSNVs (c.933A>G and c.1662G>A) [8, 11] were not observed among the sequenced samples here. As no effects would be expected by sSNVs according to theoretical analyses of splice sites, and any phenotype-genotype correlation could be excluded, these sSNVs were not further focused on in this study. Furthermore, no changes were observed at any splice sites.

**Table 2:** Observed *CMAH* gene variants (nsSNVs and indels), PROVEAN analyses, genotypes, and haplotypes in different purebred and non-pedigreed cats with blood types *A*, *B* and *C*.

Exon	DNA	Protein	Reference	PROVEAN	Genotype^a^			*A* Haplotype	*B* Haplotype			C Haplotype
					Blood type *A*	Blood type *B*	Blood type *C*		All breeds without Turkish Angora and Ragdolls	Turkish Angora	Ragdoll	Ragdoll
UTR	c.Δ-53	Unknown	[8]	Not applicable	NN,NP, PP	PP, NN	NN, NP, PP	NP	P	N	N	NP
2	c.139C>T	p.Arg47Cys	[9]	Neutral	CC, CT	CC, TT, CT	CC, CT	C	C	T	C	C
2	c.142G>A	p.Val48Met	[8]	Neutral	GG, GA, AA	AA, GG, AG	GG, GA	GA	A	G	GA	GA
2	c.179G>T	p.Gly60Val	[11]	Deleterious	GG, GT	GG, TT, GT	GG, GT	G	G	T	G	G
2	c.187A>G	p.Ile63Val	[11]	Neutral	AA, AG, GG	AA	AA, AG	AG	A	A	A	A
3	c.268T>A	p.Tyr90Asn	[8]	Deleterious	TT, TA	AA, TT, TA	TT, TA	T	A	T	TA	TA
4	c.327A>C	p.Glu109Asp	[8]	Neutral	CC, CA, AA	CC, AA	AA, AC, CC	AC	AC	C	C	AC
4	c.364C>T	p.Pro122Ser	[10]	Deleterious	CC	CC	CC, CT, TT	C	C	C	C	CT
4	c.374C>T	p.Ser125Leu	^b^	Neutral	CC	CC	CC, CT	C	C	C	C	CT
4	c.376G>A	p.Glu126Lys	^b^	Neutral	GG	GG	GG, GA	G	G	G	G	GA
5	c.593A>C	p.His198Pro	^b^	Deleterious	CC,AA	CC, AA, CA	CC, AA	CA	CA	C	CA	CA
8	c.868A>C	p.Thr290Pro	^b^	Neutral	AA	AA	AA, AC	A	A	A	A	AC
8	c.898A>G	p.Lys300Glu	^b^	Neutral	AA	AA, GG	AA, GG, GA	A	A	A	AG	AG
8	c.933delA	p.Ala312Hisfs*6	^b^	Not applicable	AA	AA, A*	AA	A	A*	A	A	A
11	c.1322delT	p.Leu441*	^b^	Not applicable	TT, T*	TT, **, T*	TT, T*	T	T	T	*	T*
11	c.1342G>A	p.Val448Ile	^b^	Neutral	GG, GA	GG, GA	GG	G	G	G	G	G
12	c.1603G>A	p.Asp535Asn	[8]	Deleterious	GG, GA	AA, GG, GA	GG, GA	G	GA	G	GA	GA

^a^ first genotype was most commonly observed;

^b^ discovered in this study; N not present, P present

### Phenotype-genotype correlations

#### Genotypes observed in type *A* cats

When comparing *CMAH* sequences obtained in this study from 14 type *A* cats of 10 breeds with the reference and other published *CMAH* gene sequences common to type *A* [8], we observed homozygosity (wildtype) and heterozygosity for six (c.139C>T, c.179G>T, c.268T>A, c.1322delT, c.1342G>A, c.1603G>A) of the 17 nsSNVs/Indels. Seven variants were present only homozygously for type *A* cats. In addition, four nsSNVs/Indels (Δ-53, c.142G>A, c.187A>G, c.327A>C) were present with all three genotypes (Table 2 and S2 Table) excluding them as being causal for blood type B. Thereby, we were able to establish 14 different diplotypes for type *A* that could be summarized in the haplotype *A* column shown in Table 2.

#### Genotypes observed in type *B* cats

When comparing the *CMAH* gene sequences obtained here from 36 type *B* cats belonging to nine breeds (plus one DSH cat) with the reference genome and the sequences of the 14 type *A* cats from this study, we found five of 17 nsSNVs/Indels (c.364C>T, c.374C>T, c.376G>A, c.868A>C, c.1342G>A) also occurring in type *A* cats indicating that they are not part of a type *B* haplotype (Table 2).

The previously published *B* variants c.142G>A (used for routinely screening of *B* cats) and c.268T>A cosegregated for all type *B* cats. Indeed, 21 type *B* cats were homozygous for both, but four type *B* cats were heterozygous and 11 were homozygous wildtype for both of these nsSNVs. However, these cats were homozygous or probably compound heterozygous for other *B* variants: six of the nine Ragdolls were homozygous for the newly discovered c.1322delT variant, and three appeared to be compound heterozygous for c.142G>A/c.268T>A and c.1322delT variants.

In addition, all four Turkish Angoras typed were homozygous for the c.179G>T variant previously described in seven cats in Japan [11]. One Neva Masquerade was probably compound heterozygous for c.142G>A/c.268T>A and c.179G>T variants and one DSH cat (from Lyon, France) was likely compound heterozygous for c.179G>T and newly discovered c.933delA variant. Moreover, the c.139C>T variant cosegregated with c.179G>T variant in all *B* cats.

Among the other observed nsSNVs, the c.187A>G variant was fixed as AA genotype in all type *B* cats, while all three genotypes were observed in the tested type *A* cats. Furthermore, the variant c.898A>G was homozygous wildtype (AA) in most cats, but three cats were homozygous for the variant (GG). All three genotypes were observed in *B* cats for the variant c.593A>C. Finally, the variant c.327A>C was seen as homozygous wildtype or variant in type *B* cats.

Furthermore, the variant c.1603G>A was previously described to cosegregate with c.268T>A and c.142G>A [8]. However, two of 36 type *B* cats in our survey showed different diplotypes for c.268T>A and C.1603G>A (one Neva Masquerade with GG at position 1603 and one Birman with GA at position 1603).

Finally, the effect of indel Δ-53 is unknown, as no CMAH protein expression studies have been performed. This indel was claimed to be associated with type *B* blood [8], but in this study, type *A* and *C* cats showed all three genotypes and type *B* cats revealed the homozygous wildtype and variant type. Thus, the indel Δ-53 did not correlate with the *B* blood type.

#### Genotypes observed in type *C (AB)* cats

All 12 type *C* Ragdolls were homozygous (*a*^*c*^*/ a*^*c*^) or heterozygous (*a*^*c*^*/b*) for the variant c.364C>T previously described as the cause of *C* type in Ragdolls [8]. Furthermore, the variant c.327A>C cosegregated in all type *C* Ragdolls with the c.364C>T variant. All type *C* Ragdolls heterozygous for the c.364C>T variant (which are also heterozygous for the c.327A>C variant) were carrying either the previously reported c.142G>A (used in screening for type *B* cats) and c.268T>A variants or the newly discovered c.1322delT variant suggesting one *B* and one *C* haplotype, i.e. the genotype *a*^*c*^*/b*. All type *C* Ragdolls homozygous for c.364C>T were also homozygous for the wildtype *A* haplotype (c.142G>A and c.268T>A variants) suggesting the genotype *a*^*c*^*/a*^*c*^.

In addition, the c.142G>A and c.268T>A variants cosegregated among all 20 type *C* cats of any other breed tested. Interestingly, the six DSH cats and one Siberian cat with type *C* blood were all heterozygous for c.139C>T, c.179G>T and c.187A>G variants (all three nsSNVs located in exon 2), but no other variants were observed to explain their *C* blood type. Of these variants only c.179G>T is considered to be deleterious and was in the homozygous state associated with type *B* in Turkish Angora cats in this study (see above, Table 2 and S2 Table). We speculate that this c.179G>T heterozygosity state may permit the expression of both blood type antigens.

Finally, there was one type *C* British Shorthair cat that was heterozygous for the c.268T>A and c.142G>A and homozygous for the Indel Δ-53 variant, but these variants did not explain a *C* blood type.

### Effects of nsSNVs on CMAH protein

While some sSNVs could still have structural and functional effects, we focused on the analysis of nsSNV/indel variants in the *CMAH* gene. The two frameshifts in the *CMAH* gene with ensuing early stop codons at p.Leu441* (c.1322delT) and p.Ala312Hisfs*6 (c.933delA) are predicted to cause mRNA decay and thus no protein or severely truncated dysfunctional enzyme proteins. Of the 14 missense variants in *CMAH*, five were called deleterious by PROVEAN analysis, while nine appeared neutral (Table 2). Concordantly, these deleterious SNVs were shown above to be associated with different blood types. Moreover, SIFT analysis predicted mostly the same consequences of the variants on protein pending parameter selection (data not shown).

### Genotyping for nsSNV c.142G>A, c.268T>A and Δ-53

Based on our sequencing results and the previously used genotyping SNVs (c.142G>A and Δ-53) we developed an additional TaqMan SNP Assay for the c.268T>A variant and screened 351 cats of different breeds with blood type *A* and *B* (Tables 1 and 3). The 241 type *A* cats were either homozygous or heterozygous for the newly introduced c.268T>A variant, but 38 of them (15.8%) showed divergent genotypes for the other two variants (c.142G>A and/or Δ-53). In fact, based on these two SNVs alone, a blood type *B* would have been predicted incorrectly for twelve cats (4.9%). In contrast, all type *A* cats in this study were homo- or heterozygous and had genotype TT or TA at position c.268 and were correctly assigned by this c.268 SNV.

**Table 3:** Diplotypes for three *CMAH* SNVs for 351 genotyped type *A* and *B* cats.

Blood type	Diplo-type	# of cats	c.142G>A	c.268T>A	Δ-53	Comments
A	A1	145	GG	TT	NN	
	A2	60	GA	TT	NP	
	A3	11	GG	TT	NP	
	A4	5	GA	TT	NN	
	A5	5	GG	TT	PP	
	A6	5	GA	TA	NN	
	A7	3	GG	TA	NN	
	A8	3	GA	TA	PP	
	A9	2	AA	TA	NN	
	A10	2	AA	TT	NN	
	A11	2	GG	TA	NP	
B	b1	90	AA	AA	PP	
	b2	2	GA	AA	PP	
	b3	1	GG	AA	PP	
	b4	1	AA	AA	NN	
	b5	1	AA	AA	NP	
	b6	10	GG	TT	NN	six Ragdolls homozygous for c.1322delT two Turkish Angora homozygous for c.179G>T one DSH homozygous for c.1193G>A one DSH remained unresolved
	b7	5	GA	TA	NP	four Ragdolls heterozygous for c.1322delT one Turkish Angora heterozygous for c.179G>T

Shaded areas mark the genotypes fitting the phenotypic blood type.

Of the 110 type *B* cats, 95 (86.4%) were homozygous for an A at position c.268. However, five of these type *B* cats (5.3%) showed divergent genotypes for the other two variants (c.142G>A and Δ-53) currently used in the genotype screening which would have predicted blood type *A*. In addition, the genotyping results of these three SNVs would have predicted a blood type *A* in 15 type *B* cats in this survey, but the cause for their type *B* blood could be explained by other variants: ten were Ragdolls and six of them were homozygous for the T deletion at position c.1322. The other four type *B* Ragdolls were heterozygous for the deletion c.1322delT as well as the c.268T>A variant suggesting compound heterozygosity.

Similarly, among the three Turkish Angora cats with type *B*, two were homozygous for the c.179G>T variant and the one heterozygous cat was also heterozygous for the c.268T>A variant suggesting again a compound heterozygous genotype. Finally, one DSH cat was homozygous for the variant c.1193G>A which result in p.Trp398*. However, for one type *B* cat no variants were identified in the exons and regulatory regions of *CMAH* which could have explained the phenotypic *B* blood.

Based upon our discoveries, we established a screening program (Table 4) with the nsSNVs/indels c.268T>A, c.179G>T, c.1322delT and c.364C>T, which consistently and accurately defined the genetic blood types of the 351 screened cats except two DSH cats with type *B*. Furthermore, including the 70 cats with sequenced *CMAH* gene, screening for those variants would have correctly assigned the blood type of all but three DSH cats with type *B*, six DSH cats with type *C* and one Siberian cat with type *C* (because c.364C>T seems to be only relevant in Ragdoll cats with type *C*). If we also included the other two discovered SNVs (c.933delA and c.1193G>A) causing type *B* blood, all 421 genotyped cats except one (type *B*) could be properly classified.

**Table 4:** Genotyping scheme with *CMAH* variants for type *A*, *B*, and *C* blood.

Blood type	c.179G>T	c.268T>A	c.364C>T	c.1322delT	Number of sequenced cats			Genotype	Breeds
					Type *A*	Type *B*	Type *C*		
*A*	GG	TT	CC	TT	9	0	0	A/A	Multiple
	GT	TT	CC	TT	1	0	1 Sibirian + 6 DSH	A/b	Turkish Angora
	GG	TA	CC	TT	3	0	1 BSH	A/b	All breeds
	GG	TT	CC	T*	1	0	0	A/b	Ragdoll
*B*	TT	TT	CC	TT	0	4	0	b/b	Turkish Angora
	GG	AA	CC	TT	0	20	0	b/b	Multiple
	GG	TT	CC	**	0	6	0	b/b	Ragdoll
	GT	TA	CC	TT	0	1	0	b/b	Neva Masquerade
	GT	TT	CC	T*	0	0	0	b/b	#
	GG	TA	CC	T*	0	3	0	b/b	Ragdoll
	GG	AA	CC	T*	0	1	0	b/b	Scottish Fold
*C*	GG	TT	TT	TT	0	0	3	a^c^/a^c^	Ragdoll
	GG	TA	CT	TT	0	0	5	a^c^/b	Ragdoll
	GG	TT	CT	T*	0	0	4	a^c^/b	Ragdoll

Shaded areas highlight the relevant genotypes: dark grey represents homozygous for non-A variant/allele, light grey represents the heterozygotes. # are the theoretical possibilities.

## Discussion

Since Landsteiner’s seminal discovery of the ABO blood group system, the discovery and diagnostic blood typing in humans and animals has been pursued by RBC surface antigen to alloantibody interactions [1, 2]. Polyclonal (antisera) and later monoclonal antibodies have been principally used in hemagglutination and other immunohematological assays (forward typing). If naturally or induced alloantibodies are present, back typing with RBCs of known type can substantiate the test results [3]. To that end, we utilized an agglutination gel column laboratory method with *anti-B* lectin and feline *anti-A* serum and immunochromatographic strip kit with murine *anti-A* and *anti-B* monoclonal antibodies to type 421 domestic cats for blood type *A*, *B*, and *C* (usually called *AB* type). The resulting blood types were completely concordant, similarly accurate to prior feline typing surveys with these methods but better compared to card and cartridge hemagglutination kits.

While immunohematological assays are simple, practical, and accurate, when properly performed, they have, beside obvious sample quality and operator related problems, various limitations related to the strength and specificity of alloantibody and RBC antigen leading to typing inconsistences and inaccuracies. Weaker RBC antigens may not be consistently identified in complicated clinical situations due to prior transfusions and autoantibodies, and recessive blood type alleles cannot be detected in the heterozygous state [12–14]. Therefore, molecular approaches that are capable of detecting DNA variants underlying the phenotypic blood type have been established to complement immunohematological methods as diagnostic tools in humans [19, 20]. They cannot resolve all complex clinical situations, but these methods are readily standardized as well as automated and lend themselves to high throughput.

While in humans *CMAH* is a pseudogene due to an universal ancestral large deletion, the CMAH enzyme, when functional, converts NeuAc (type *B* antigen) to NeuGc (type *A*) in domestic cats [3]. In limited prior studies, several SNVs have been found in the feline *CMAH* gene, and a few variants, without showing their deleterious effects, were thought to be related to blood type *B* and *C*. Furthermore, when genotyping cats with those variants, blood typing results showed inconsistent phenotype to genotype correlations. In this large survey of 421 carefully blood typed cats, we demonstrate few specific *CMAH* variants, predicted to be deleterious by PROVEAN, to be responsible for blood type *B* and *C*, and describe a novel genotyping scheme to accurately determine blood type *A*, *B*, and *C* in cats.

In this study we show that the variant c.268T>A was homozygously present in 86% of all type *B* cats. The remaining type *B* cats were either homozygous for the variants c.179G>T and c.1322delT or compound heterozygous for two of these three SNVs. Genotyping these three SNVs leads to an excellent screening method to molecularly type cats for blood type *A* and *B* (99%). Thereby, we propose a genotyping scheme with four variants c.179C>T, c.268T>A, c.1322delT (for blood type *B*) and c.364C>T to include the most common variant for blood type *C* (Table 4).

Homozygosity or heterozygosity of the wildtype T at position c.268 (p.90Tyr) consistently predicted blood type *A* as expected for a dominant trait. The c.268T codes for non-polar tyrosine in the functional domain of the CMAH enzyme (Rieske-domain), while the variant A at position c.268 results in a polar asparagine acid (p.90Asn) which is predicted to be deleterious and to cause enzymatic dysfunction. And indeed, p.90Asn homozygosity (genotype *b/b*) explained blood type *B* in 86% of typed cats in this study. In contrast, the previously reported nsSNV c.142G>A [8] is predicted to be a neutral valine to methionine (Val48Met) exchange, and while frequently co-segregating, it also was found to be non-predictive in 15.6% of the cats studied here. In fact the missense variant c.142G>A (p.Val48Met), which was proposed to be a DNA diagnostic marker for type *B* blood [8], is predicted to be neutral by PROVEAN and was indeed observed with all three blood types in this study. Thus, determining the c.268T>A nsSNV (p.Tyr90Asn), and not c.142G>A, is most critical in screening and differentiating type *A* and *B* cats. Other nsSNVs seen in type *A* cats in this study either co-segregated with c.268T and were neutral or could be attributed to diplotypes causing type *B* blood when homozygous or compound heterozygous.

The previously reported [11] but ignored variant c.179G>T (p.Gly60Val) and newly discovered c.1322delT (p.Leu441*) variant in *CMAH* are predicted to be deleterious and are shown here to also result in type *B* blood when homozygous (*b/b*) for either in Turkish Angora (and thus likely Turkish Van) and Ragdoll cats, respectively. Furthermore, some DSH cats (e.g. from Israel) were either also homozygous for c.179T variant or were probably compound heterozygotes in combination with the c.268A variant. All type *B* Ragdolls were either homozygous c.268A or c.1322delT or were likely compound heterozygotes at these two positions. Thus, when screening cats by combining the missense variants c.268T>A and c.179G>T and nonsense variant c.1322delT, we were able to genotypically classify 99.5% of the 351 screened type *A* and *B* cats; missing two type *B* cats with different variants (see below).

Based upon these analyses, the previously described [10] missense variant c.364C>T (p.Pro122Ser) is predicted to be deleterious, and diminishes CMAH activity permitting both *A* and *B* antigen expression in 12 homozygous (*a*^*c*^*/ a*^*c*^) type *C* Ragdolls. All nine heterozygous type *C* Ragdolls (genotype *a*^*c*^*/b*) showed a heterozygous state at either position c.268 or c.1322. Furthermore, we propose that c.179G>T (Gly60Val) in a heterozygous state may have impaired CMAH activity and led to blood type *C* as seen in six DSH cats and one Siberian cat as seen in this study here.

Thus, including the c.364C>T (p.Pro122Ser) in a genotyping screening program will identify many but not all *C* type cats and should be combined with testing for c.268T>A (p.Tyr90Asn), c.1322delT (p.Leu441*), and c.179G>T (Gly60Val) (Table 4). The initial step is the variant c.268T>A, because the c.268T>A variant (genotype AA) was responsible for the vast majority of type *B* cats (86%). Heterozygous cats with genotype TA at position 268 may have blood type *A* and simply carry the *b* allele or have type *B* or *C* blood based upon other variants shown above or to be determined.

Homozygosity for the deletion c.1322delT can be responsible for type *B* alone or as a compound heterozygote with c.268T>A. Determination of just these two variants permitted the correct identification of all type *A* and *B* Ragdolls but not type *C* Ragdolls.

Homozygosity at nsSNV c.179 (genotype TT) also causes type *B* as shown here in Turkish Angora (and likely related Van) cats as well as related Neva Masquerade but also type *B* DSH cats from Israel and Turkish DSH cats (unpublished).

Homozygosity for T at position c.364 is not the only genotype associated with type *C*, compound heterozygotes with c.364CT and c.268TA or c.179CT may also result in type *C* in Ragdolls and other breeds of cats.

Unfortunately, in vitro gene expression studies were neither performed nor in previously published studies. Blood type relates directly to the expression of NeuAc and/or NeuGc which was shown in our prior studies by thin layer chromatography and flow cytometry [6]. Furthermore, we used three typing methods to assure proper classification or RBC antigen expression. However, we did not perform further biochemical and gene expression studies to directly document the reduced enzymatic CMAH activity and did not perform crystallographic and other structural analyses of CMAH.

In feline medicine, genotyping for the *AB* blood group system is highly desirable to confirm the rare *B* and *C* blood types, to assure *A-B* compatible transfusions, and to determine if a type *A* cat carries one *b* (*A/b*) or *a*^*c*^ (*A/a*^*c*^*)* allele for breeding. Based upon our identification of deleterious genetic variants in the *CMAH* gene and correlation to the phenotypic *A*, *B*, and *C* blood types, we propose, in combination with immunohematological typing, a novel simple genotyping algorithm which may be expanded when additional variants are identified as causes of type *B* and *C* in certain breeds and geographic regions. The proposed algorithm for determining blood types *A*, *B*, and *C* can be readily standardized and automated by software programs to assure technical as well as blood type calling accuracy. However, finding additional unique variants in certain breeds and geographic regions, as already shown here with this study, also illustrates that other variants may be found causing type *B* and *C* and thus the algorithm may need to be expanded in the future. This should not be difficult considering the ease of multiplexing and expanding existing panels.

In summary, we sequenced the *CMAH* coding and regulatory region of 70 cats and genotyped additional 351 carefully blood typed purebred and non-pedigreed cats and document novel variants predicting hydrolase dysfunction and unique haplotypes to explain their blood type *A*, *B*, and *C*. Thereby, a new diagnostic scheme to genotype all cats for the *AB* blood group system differentiating types *A*, *B*, and *C* was shown to be 99% accurate and is proposed to replace prior genotyping schemes. Furthermore, additional genetic variants responsible for type *A*, *B*, and *C* in unique breeds or geographical regions could be simply added to this genotyping scheme.

## Supporting information

S1 Fig All found variants and their location in feline *CMAH* gene. (EPS)

S1 Table Primers used for CMAH sequencing. (DOCX)

S2 Table All observed *CMAH* gene variants in sequenced cats separated in nsSNVs and sSNVs. (XLSX)
