# Supplementary material for: Molecular characterization of blood type A, B, and C (AB) in domestic cats and a CMAH genotyping scheme
Source: PLoS One. 2018 Sep 20;13(9):e0204287. doi: 10.1371/journal.pone.0204287 (PMC6147723; doi:10.1371/journal.pone.0204287)
Supplement: S1 Table — (DOCX) [file pone.0204287.s002.docx]

S1 Table. Primers used for CMAH sequencing

| Exon 2 Forward | TGT AAA ACG ACG GCC AGT TGA GCA AGC AGA GCG TGC AT |
| --- | --- |
| Exon 2 Reverse | GCA GGA AAC AGC TAT GAC AAG TGA GCA CAC ACG AAG ATT ATG |
| Exon 3 Forward | TGT AAA ACG ACG GCC AGT TGA AGT GGT GCT CAA GCC CCT CAT TTA |
| Exon 3 Reverse | GCA GGA AAC AGC TAT GAC CTG GTC ACA GTC TTG TCC TAG GCA C |
| Exon 4 Forward | TGT AAA ACG ACG GCC AGT TGA CCC TTT GAT TTT TGG TAC TCT GTG TAC |
| Exon 4 Reverse | GCA GGA AAC AGC TAT GAC CTA ATA CAC ACA CAT AAA TGG ACA TCT |
| Exon 5 Forward | TGT AAA ACG ACG GCC AGT TGA TAA AAC CAA CCG GGA GAG TTT CTA T |
| Exon 5 Reverse | GCA GGA AAC AGC TAT GAC CAC ACA TGA AAA CTG TAT GAT ATT CGC |
| Exon 6-7 Forward | TGT AAA ACG ACG GCC AGT TGA GGA GCA AAC TGT CTC TTC TAC TAG GTG |
| Exon 6-7 Reverse | GCA GGA AAC AGC TAT GAC CGA AAT GAA GAT AGT AGA AGA TGG TTA |
| Exon 8 Forward | TGT AAA ACG ACG GCC AGT TGA TTC CTT GGA TAG AAG TAA TTC TTC TCT |
| Exon 8 Reverse | GCA GGA AAC AGC TAT GAC CGT TTC CCA GTT AAG ACT GTA AAC TTC |
| Exon 9 Forward | TGT AAA ACG ACG GCC AGT TGA AGA GAG CAC TTT CTA CTC ATG ACA CA |
| Exon 9 Reverse | GCA GGA AAC AGC TAT GAC CAG ACA ATC TCT CTG AAT GGA TTC TAG |
| Exon 10 Forward | TGT AAA ACG ACG GCC AGT TGA AAG ACA AAG TAT AAG TAC AAC TTC TTG |
| Exon 10 Reverse | GCA GGA AAC AGC TAT GACCAC AAA GAC TTA ATA TTC CTA CAA ACG |
| Exon 11 Forward | TGT AAA ACG ACG GCC AGT TGA CAA AAG ATG TGG AGT TTT CCC ATA GT |
| Exon 11 Reverse | GCA GGA AAC AGC TAT GAC CAA CAA TCG TGG GAC AAG AAT GCT |
| Exon 12 Forward | TGT AAA ACG ACG GCC AGT TGA GAG GGA CTA TAC CGA ACT CCA ATT CAT T |
| Exon 12 Reverse | GCA GGA AAC AGC TAT GAC CGA CAA CTA GCA CAG TAA AGG AAG GTG |
| Exon 13 Forward | TGT AAA ACG ACG GCC AGT TGA TGC GTT CTG TAG ATG AGG AGA CTG C |
| Exon 13 Reverse | GCA GGA AAC AGC TAT GAC CGA ATG AAG CTC TAA TGG TGG AAT CT |
| Exon 14 Forward | TGT AAA ACG ACG GCC AGT TGA AAA TGC CAG GCA CTG AAA CAG TA |
| Exon 14 Reverse | GCA GGA AAC AGC TAT GAC CGA TTT AGC TCT GAA CAC TCA CTG CC |
| Exon 15 3´-UTR Forward | TGT AAA ACG ACG GCC AGT TGA CGT ATA GTT CAG |
| Exon 15 3´-UTR Reverse | GCA GGA AAC AGC TAT GAC CCC TGA CTC TCT |
| Exon 1a Forward | TGT AAA ACG ACG GCC AGT TGA AAT TTA CAG |
| Exon 1a Reverse | GCA GGA AAC AGC TAT GAC CTA GCA AAC ATA |
| 1b Forward | TGT AAA ACG ACG GCC AGT TGA CGC TCT CCT TGC TCA GTG TTG |
| 1b Reverse | GCA GGA AAC AGC TAT GAC CCA ACA CTG AGC |
